# Scout In, Scout out: Savi scout reflector traversing a dilated duct to the nipple in breast cancer localisation—a case report

**DOI:** 10.1093/omcr/omae196

**Published:** 2025-03-20

**Authors:** Aman Saswat Sahoo, Monther Salman, Bhuvi Singh, Gina Weston-Petrides, Lilia Ragad, Rasheed Elayyan

**Affiliations:** Fylde Rd, School of Medicine and Dentistry, University of Central Lancashire, Preston PR1 2HE, The United Kingdom; Breast Surgery Department, King’s College Hospital, Denmark Hill, London SE5 9RS, The United Kingdom; Fylde Rd, School of Medicine and Dentistry, University of Central Lancashire, Preston PR1 2HE, The United Kingdom; Breast Surgery Department, King’s College Hospital, Denmark Hill, London SE5 9RS, The United Kingdom; Fylde Rd, School of Medicine and Dentistry, University of Central Lancashire, Preston PR1 2HE, The United Kingdom; Breast Surgery Department, King’s College Hospital, Denmark Hill, London SE5 9RS, The United Kingdom; Breast Surgery Department, King’s College Hospital, Denmark Hill, London SE5 9RS, The United Kingdom; Breast Surgery Department, King’s College Hospital, Denmark Hill, London SE5 9RS, The United Kingdom; Breast Surgery Department, King’s College Hospital, Denmark Hill, London SE5 9RS, The United Kingdom

**Keywords:** breast cancer, wide local excision, savi scout, localisation, invasive ductal carcinoma

## Abstract

Introduction: Savi scout system is being widely used for localising and excising breast tumours. While the migration of scout reflectors has been documented, this is the first case of a Savi Scout reflector migrating through a dilated duct near the lesion and coming out of the nipple. Case Presentation: A 56-year-old postmenopausal woman with a history of right breast intraductal papilloma which transformed to Grade II Invasive Ductal Carcinoma (IDC) has a Savi Scout reflector placed in the tumour. However, it migrated through a dilated duct and emerged at the nipple, causing severe pain. The reflector was then surgically removed, and the patient subsequently underwent wide local excision with skin marker localisation. Conclusion: Anatomical variations such as presence of dilated ducts need to be considered before placing scout reflectors. Appropriate positioning would prevent them from migrating through such ducts avoiding patient discomfort and further procedures for localisation.

## Introduction

Novel techniques to facilitate precise localisation for wide local excision include the Savi Scout (SS) system, which has been used since 2014. A small radar-activated reflector measuring 12 × 1.6 mm is placed percutaneously in the lesion under image guidance. A handheld probe is used to identify the lesion margins and allows the surgeon to easily excise the tumour [1].

From a patient’s perspective, the SS reflector has no external wires placed through the skin, further enhancing patient experience. It is associated with common complications like bleeding and infection. There have been documented instances of both minor and significant migration of the reflector. However, most literature reports no complications with the use of SS.

Herein, we present a case involving a patient with an impalpable invasive cancer of the breast who underwent placement of a SS reflector within the tumour. However, the reflector migrated from the tumour, traversed a dilated duct, and eventually came out through the nipple. The first of its kind.

## Case presentation

A 56-year-old postmenopausal woman presented with a history of intermittent mastalgia, mild lumpiness, and clear nipple discharge of the right breast, which started a year ago. She has a previous known history of right breast intraductal papilloma without atypia 8 years ago, for which she refused the intervention at that time. Her mammogram two years ago was reassuring.

**Figure 1:**
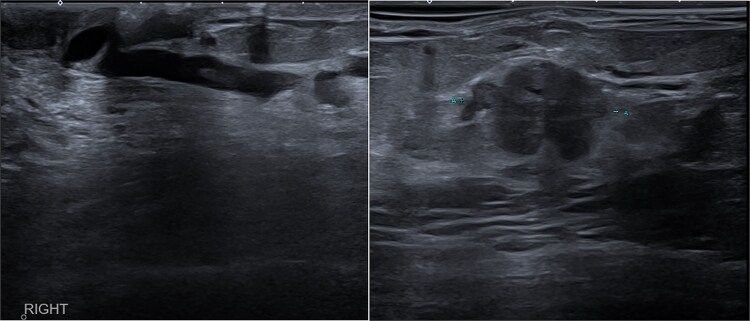
Initial USS of the dilated duct of Rt breast (left), initial USS of IDC lesion in Rt breast (right).

An ultrasound scan revealed a highly suspicious mass at the 2 o’clock position of the right breast periareolar area, measuring 17 mm, with a hypoechoic dilated duct measuring over 50 mm in length and 3.4 mm in diameter at the medial aspect of the right nipple (Fig. 1). The axilla appeared normal on the ultrasound scan.

**Figure 2:**
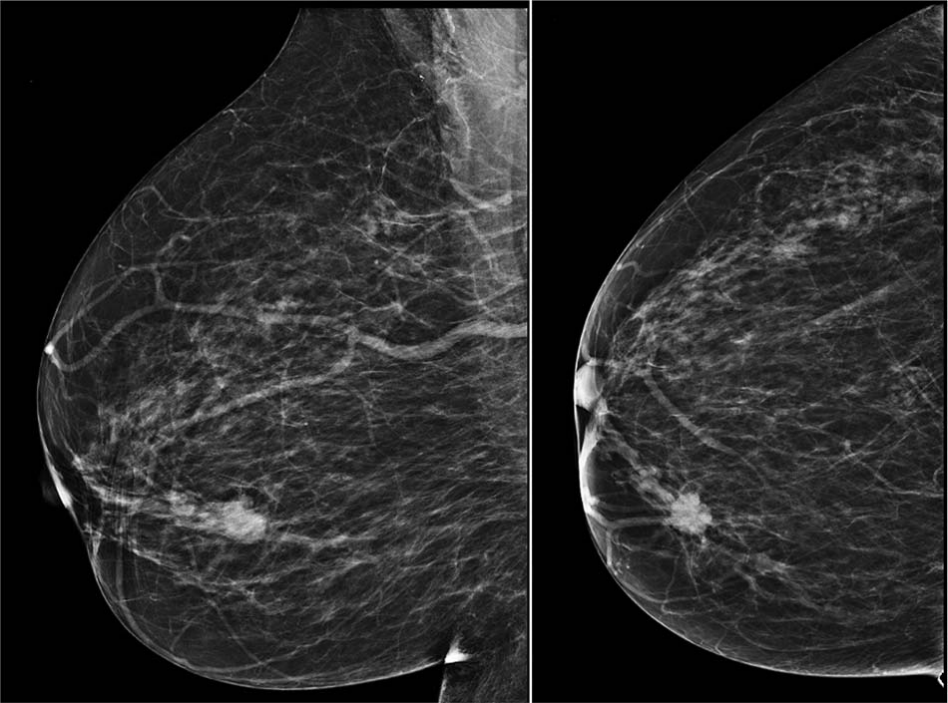
Mammogram of the lesion in breast RMLO (left) and RCC (right).

Upon comparison of mammograms from eight years ago and two years ago, the lesion was identified as a new development (Fig. 2). An ultrasound-guided core biopsy of the right breast mass was performed. The histopathological evaluation revealed a Grade II Invasive Ductal Carcinoma.

**Figure 3:**
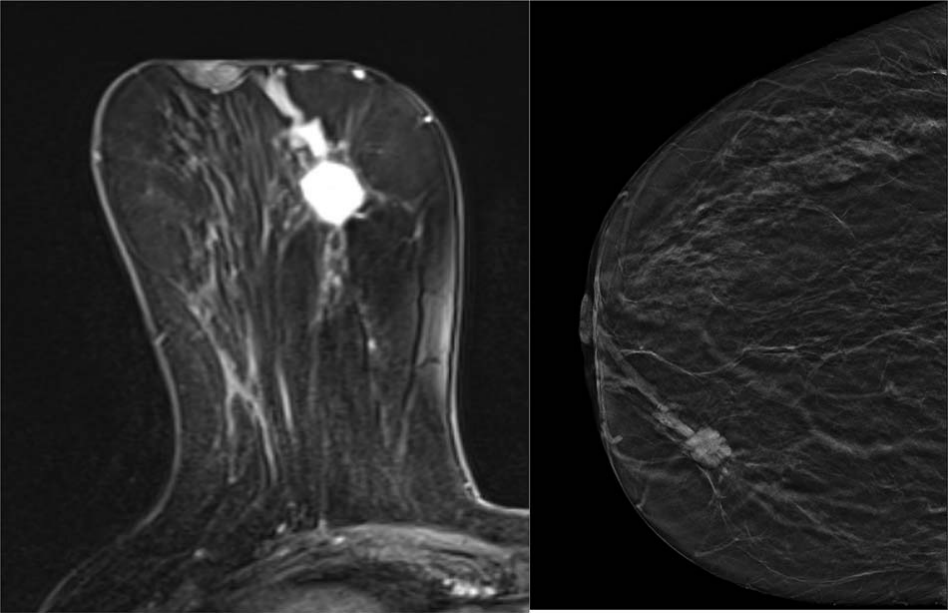
MRI of Rt breast (left), Tomosynthesis of Rt breast (right).

After a multidisciplinary team meeting, a Magnetic Resonance Imaging (MRI) and breast tomosynthesis were ordered to assess the disease extent (Fig. 3). The known malignancy of the upper inner quadrant was identified, measuring 26 mm in the longest dimension. The MRI study identified an anterior extension accounting for an overall extent of 31 mm.

**Figure 4:**
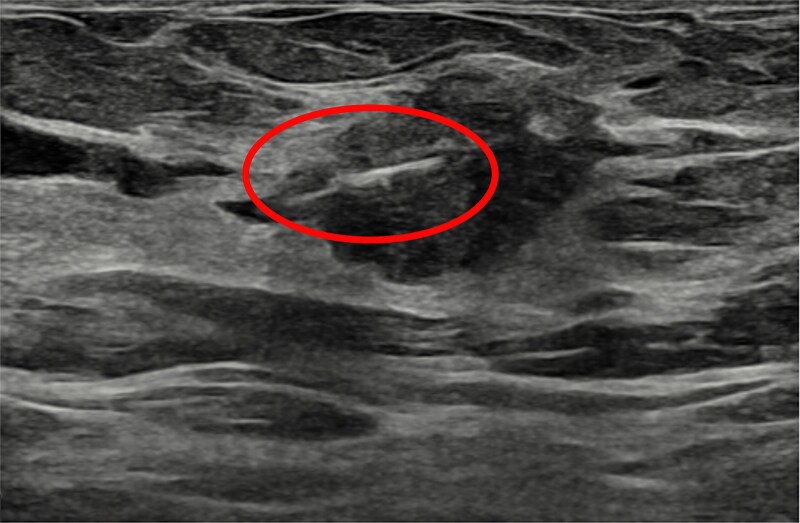
USS showing Savi scout placed in the lesion.

A SS reflector was placed at the site of the tumour under ultrasound guidance (Fig. 4). No immediate complications were noted. Post-insertion mammogram has been obtained.

**Figure 5:**
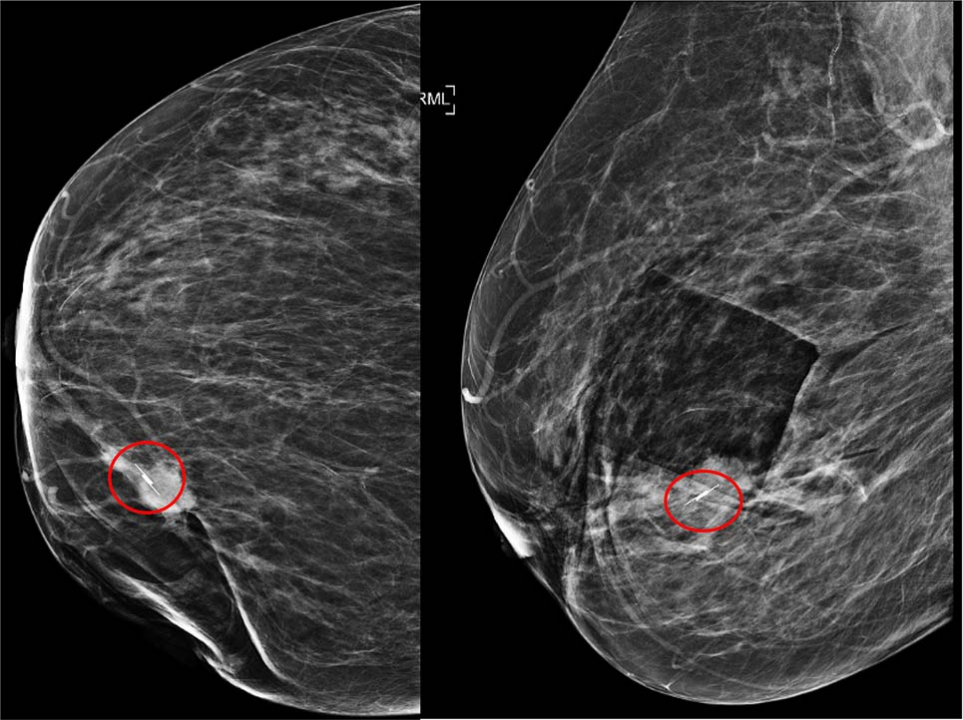
Mammogram post Savi scout (circled) insertion RCC view (left), mammogram post Savi scout insertion (circled) RLMO view (right).

Imaging revealed that the scout was successfully placed in the lesion (Fig. 5).

**Figure 6:**
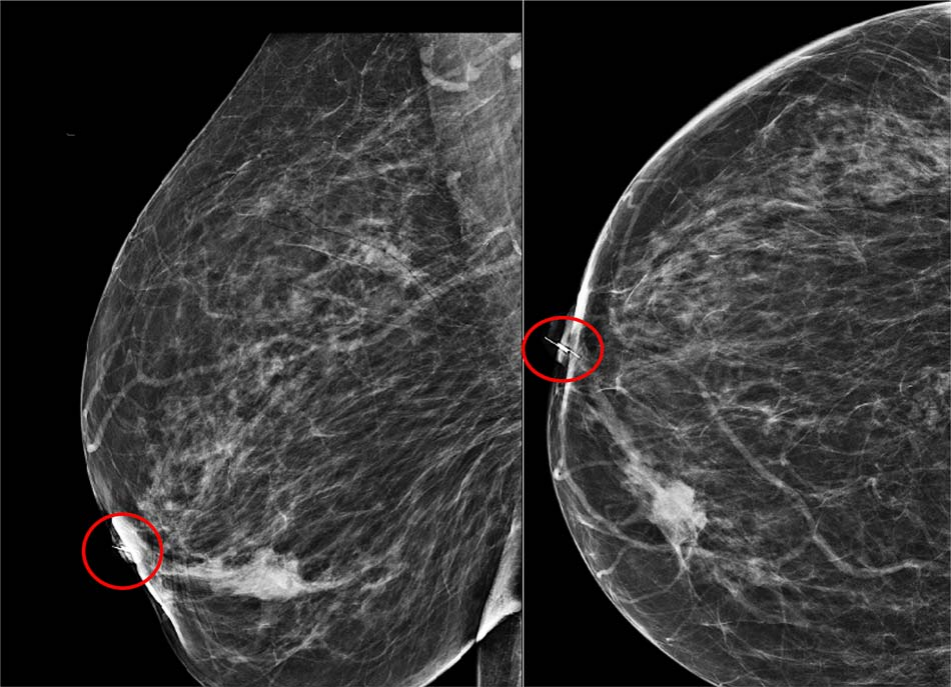
Mammogram of migrated Savi scout RMLO view (left), mammogram of migrated Savi scout RCC view (right).

Two days later, the patient presented to the breast clinic complaining of severe stabbing pain in the right nipple. An ultrasound and mammogram scan revealed that the scout had migrated from the site of the cancer into the duct at the tip of the nipple (Fig. 6).

Under local anaesthesia, a small incision was made on the skin over the tip of the scout, and it was removed. The patient visited the clinic the next day and reported reduced pain and discomfort since the scout was removed.

The patient declined to have another SS reflector due to distress during the initial procedure. The decision has been made to proceed with skin marker-guided wide local excision, which she has had. Postoperative histopathological evaluation showed 27 mm grade 2 invasive ductal carcinoma (IDC) no special type, with no associated ductal carcinoma in situ, which is completely excised, and sentinel node biopsy was negative.

## Discussion

This case describes an unreported complication where the SS migrated through a dilated duct to the nipple. The migration is attributed to a dilated duct with a diameter of 3.4 mm, large enough for the 1.6 mm wide device to traverse (Fig. 1). Bhattarai et al. [2] demonstrated the association between ductal papilloma and ductal dilation, with ducts ranging from 2 to 10 mm in diameter.

The American Society of Breast Surgeons [3] recommends excising papillomas due to their malignancy risk, as 67% of papillary lesions with atypia are upgraded upon excision. In this case, an intraductal papilloma may have progressed to invasive ductal carcinoma.

Ultrasound and mammogram images showed the tumour adjacent to a dilated duct, emphasizing the importance of careful device placement. Kramer et al. [4] highlight that tomosynthesis-guided Scout reflector placement with a lateral arm attachment improves control, precision, and efficiency by allowing single-view procedures, reducing radiation and time.

Hayes et al. [5] reported a 1% migration rate with SS with potential complications such as pain, infection, and non-functional devices Mango et al. [6] noted migration in one of fifteen cases due to post-biopsy hematoma, though no reports exist of SS migration to the nipple. In a study with 72 SS reflectors, the reported migration rate was 0% [7]. Magseed, an alternative, has a twisted design which promotes tissue in-growth which anchors it firmly reducing chances of migration. In a study of 1123 Magseeds only 2.5% migrated [8]. LOCalizer, another alternative, in a study of 848 patients had a migration rate of 0.7% [9]. There is no statistical difference between the migration rate of all these devices. Wire localisation has a re-positioning rate of 4% [10] and these wire-free methods are a significant improvement. The SS is preferred for patients undergoing neoadjuvant chemotherapy as it uses infrared signals which do not generate MRI artifacts unlike the Magseed. This allows earlier deployment of SS in patients. However, it may fail to generate signals in deeply located lesions.

This case highlights the need for attention when placing localization devices near dilated ducts. However, there are no reports of migration of the SS reflector from the original tumour site to the nipple.

## Conclusion

The primary takeaway is the role of imaging, identification of ducts and precise localisation, considering structural anatomy, to minimise the migration of the SS reflector or other localisation devices, moreover, importance of excising papillary lesions to prevent future malignant transformations and structural changes.
